# Early structural lung changes in young children with cystic fibrosis: a call to action

**DOI:** 10.36416/1806-3756/e20250402

**Published:** 2026-05-22

**Authors:** Aline da Silva Amoras, Tália Andrea Soria Muñoz, Leticia Cole de Melo, Silvia Onoda Tomikawa, Miriam Cardoso Neves Eller, Cleyde Myriam Aversa Nakaie, Joaquim Carlos Rodrigues, Luiz Vicente Ribeiro Ferreira da Silva-Filho

**Affiliations:** 1. Unidade de Pneumologia Pediátrica, Instituto da Criança e do Adolescente, Hospital das Clínicas, Faculdade de Medicina, Universidade de São Paulo - HCFMUSP - São Paulo (SP) Brasil.

**Keywords:** Cystic fibrosis, Child, preschool, Tomography, X-ray computed

## Abstract

**Objective:**

Cystic fibrosis (CF) lung disease begins in early childhood, despite close follow-up and monitoring. Although imaging methods such as chest CT may detect structural changes before significant clinical manifestations, their role in preschool children remains debated. Evidence from low- and middle-income countries is limited. We sought to investigate the age at first chest CT, as well as imaging findings and their impact on treatment decisions.

**Methods:**

We conducted a retrospective cross-sectional study including children with CF attending a referral center in the city of São Paulo, Brazil, between 2009 and 2019. Clinical data were obtained from patient medical records and the Brazilian CF Registry database.

**Results:**

A total of 90 children were included, of whom 56.7% were male and 71.1% carried at least one F508del variant. The median age at CF diagnosis was 2.4 months (IQR, 1.5-4.5). The first chest CT was performed at a median age of 36.7 months (IQR, 28.1-47.8). Structural lung abnormalities were common, including air trapping (in 86.6% of the children), mucoid impaction (in 75.5%), bronchiectasis (in 45.5%), and atelectasis (in 35.5%), with only 4.4% of the scans being considered normal. Prescription of dornase alfa was based on CT findings in 74% of the cases, with initiation at a median age of 36.9 months (IQR, 28.9-57.6). A strong correlation was observed between age at first chest CT and age at initiation of dornase alfa therapy (r = 0.658; p < 0.001).

**Conclusions:**

Early chest CT revealed a high prevalence of structural lung disease in Brazilian infants and preschool children with CF and strongly influenced the initiation of dornase alfa.

## INTRODUCTION

Cystic fibrosis (CF) is an autosomal recessive genetic disease resulting from alterations in the expression and/or function of the CF transmembrane conductance regulator (CFTR) protein, which is a chloride and bicarbonate channel expressed in most epithelial cells.^1^ In the respiratory system, CFTR dysfunction leads to mucus accumulation, recurrent and chronic infections, and an inflammatory process that causes progressive structural changes in the airways.^1^ Pulmonary manifestations of CF often appear in early childhood and vary in severity, thus making accurate characterization difficult.^2^ The use of imaging exams such as chest CT in early childhood is controversial, and pulmonary function tests are difficult to perform in this age group.^3^ ^,^ ^4^

Structural lung changes, including bronchiectasis, have been detected in infants with CF (mean age, 3 months) diagnosed through newborn screening, being commonly associated with neutrophilic inflammation and elastase activity, even in the absence of symptoms.^5^ In high-income countries, cohort studies of children diagnosed through CF newborn screening show that structural lung abnormalities may be present from infancy and persist into the preschool years, although they are not always consistent over time.^4^ Therapeutic resources for treating lung disease in this age group are also limited and are associated with a significant daily burden for families. The use of inhaled 7% hypertonic saline has shown favorable evidence for functional and imaging outcomes in children with CF in the 3- to 6-year age bracket.^6^ ^,^ ^7^ However, data on the use of dornase alfa in this age group are practically nonexistent. 

Because an increasing number of patients with CF have received highly effective CFTR modulator therapies, particularly at younger ages, it is essential to have tools capable of detecting mild lung disease and identifying subtle changes in disease status. Ongoing research is aimed at improving follow-up strategies and developing sensitive imaging methods capable of detecting early structural lung changes in patients with CF, supporting timely intervention and monitoring disease progression.^8^ ^,^ ^9^

Most of the available evidence on early structural lung disease in patients with CF comes from high-income countries, and there is a scarcity of real-world data from middle-income settings such as Brazil, where diagnostic protocols, access to imaging, and therapeutic resources may vary substantially. In 2010, with the implementation of the CF newborn screening program in the state of São Paulo, an outpatient clinic was established in our center for the care of infants and preschool children with CF. However, there is currently no protocol determining at what age or how often patients should undergo chest CT scans for the assessment of CF lung disease at our institution. Typically, the first scan is performed when patients are 2-3 years of age, being guided by individual clinical presentation or abnormalities seen on chest X-rays. 

The objective of the present study was to determine the mean age at which the first chest CT scan was performed in children with CF attending our center, as well as to describe the main CT findings and investigate their relationship with dornase alfa prescription. 

## METHODS

This was a retrospective observational cross-sectional study conducted at a pediatric CF center in the city of São Paulo, Brazil. The center provides care for public and private patients. We reviewed the medical records and imaging findings of all CF patients in the 0- to 5-year age bracket attending our outpatient clinic between 2009 and 2019. The diagnosis of CF was established in accordance with international consensus criteria, being based on newborn screening, sweat chloride testing, and/or genetic analysis. 

The primary outcome was the presence of structural lung abnormalities on the first chest CT scan, including air trapping, mucoid impaction, bronchiectasis, and atelectasis. Clinical, laboratory, and demographic data were obtained from patient medical records and the Brazilian CF Registry, including sex, genotype (presence of the F508del variant), nutritional status (BMI Z-score), and microbiological profile. Nutritional and microbiological data were collected for the same year in which the first chest CT scan was performed. 

The scans were performed either at the center or at affiliated imaging centers, being evaluated by our pediatric pulmonology and radiology teams as part of routine clinical practice. No standardized radiological scoring systems were used. 

For analytical purposes, inhaled dornase alfa prescription was considered to be associated with CT findings when documented in the medical record within 180 days after the CT scan. This time frame was defined to reflect health care delivery in the public health care system, including the time required for imaging report availability and scheduling follow-up visits. 

Statistical analyses included descriptive statistics for continuous variables, presented as means and standard deviations or medians and interquartile ranges. The association between age at first chest CT and age at initiation of dornase alfa therapy was assessed with Spearman’s correlation coefficient. The level of significance was set at α = 0.01. All analyses were performed with the IBM SPSS Statistics software package, version 20.0 for Macintosh (IBM Corporation, Armonk, NY, USA). 

## RESULTS

Data from 90 infants with CF were analyzed, and the demographic characteristics are presented in Table 1. The median age at diagnosis was 2.4 months (IQR, 1.5-4.5), and 51 patients were male. 

**Table 1:** Demographic characteristics of the cystic fibrosis patients included in the present study (N = 90).^a^

Age at diagnosis, months	
	2.4 [1.5-4.5]
Sex	
Male	51 (56.7)
Female	39 (43.3)
Race and ethnicity	
White	75 (83.3)
Black	3 (3.3)
Mixed^b^	12 (13.3)
Genetics (F508del variant)	
Homozygous	30 (33.3)
Heterozygous	34 (37.7)
Other variants (No F508del)	26 (28.8)
Meconium ileus at birth	13 (14.4)
Pancreatic function	
Pancreatic insufficient	80 (88.9)
Pancreatic sufficient	10 (11.1)
Sweat chloride (mEq/L)^c^	
	93.13 ± 15.69
Colonization (in the year in which the first CT scan was performed)	
*Staphylococcus aureus*	64 (71.1)
*Pseudomonas aeruginosa*	42 (46.6)
Mucoid *Pseudomonas aeruginosa*	9 (10.0)
MRSA	11 (12.2)
*Burkholderia cepacia* complex	18 (20.0)

MRSA: methicillin-resistant *Staphylococcus* aureus. ^a^Data presented as n (%), mean ± SD, or median [IQR]. ^b^Black and White ancestry. ^c^Only cystic fibrosis patients with sweat tests performed via coulometry were included; the remaining patients underwent sweat testing via conductivity measurement.

**Figure 1:**
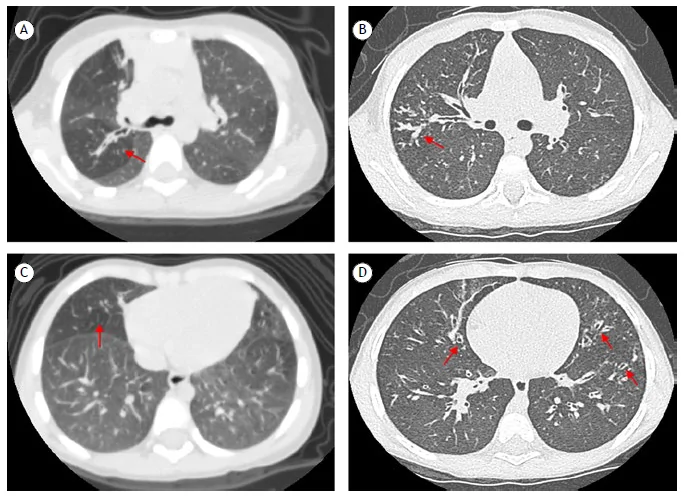
Chest CT findings in two pediatric cystic fibrosis patients at 3 years of age. In A, regional air trapping and bronchiectasis. In B, middle lobe air trapping. In C, mucoid impaction. In D, bronchiectasis. All findings are indicated by red arrows.

As can be seen in Table 2, the median age at first chest CT scan was 36.7 months (IQR, 28.1-47.8). The most common chest CT finding was regional air trapping (a mosaic pattern), in 78 patients (86.6%), followed by mucoid impaction, in 68 (75.5%), bronchiectasis, in 41 (45.5%), and atelectasis, in 32 (35.5%). Only 4 scans (4.4%) were considered completely normal (Table 2). Examples of chest CT findings are presented in Figure 1. 

**Table 2:** Chest CT findings and age at initiation of dornase alfa in the cystic fibrosis patients included in the present study (N = 90).^a^

Age at first CT scan, months	
	36.7 [28.1-47.8]
Findings on the first CT scan	
Air trapping	78 (86.6%)
Mucoid impaction	68 (75.5%)
Bronchiectasis	41 (45.5%)
Atelectasis	32 (35.5%)
Normal findings	4 (4.4%)
Age at initiation of dornase alfa, months	
	36.9 [28.9-57.6]

a Data presented as n (%) or median [IQR].

Dornase alfa was initiated in 84 patients, with a median age of 36.9 months (IQR, 28.9-57.6). In 62 (73.8%), dornase alfa was initiated on the basis of chest CT findings. In 12 (14.2%), chest CT findings did not lead to initiation of dornase alfa, and in 10 (11.7%), dornase alfa was started before the first chest CT scan was performed, for clinical reasons. 

**Figure 2:**
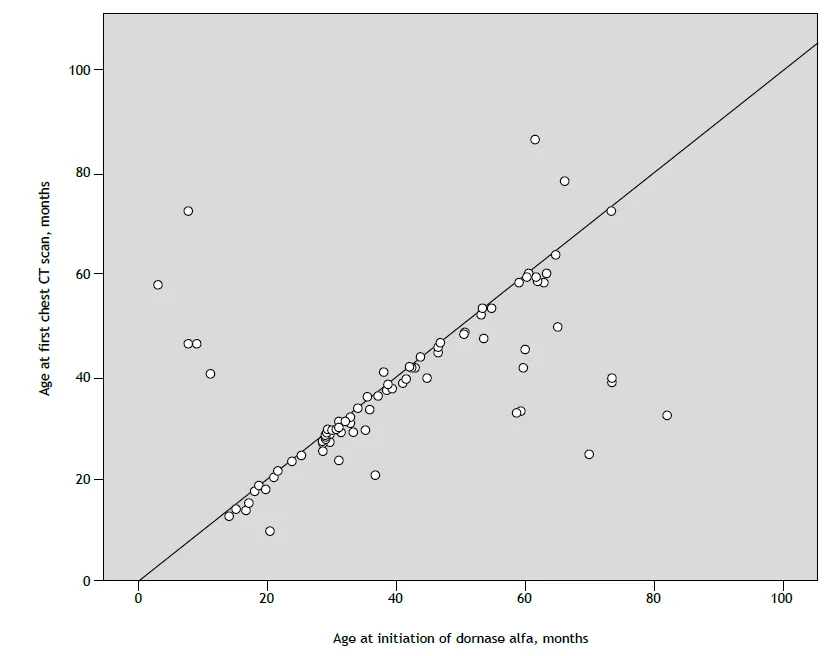
Correlation between age at first chest CT scan and age at initiation of dornase alfa (N = 90). r = 0.658; p < 0.001.

As can be seen in Figure 2, there was a strong correlation between the age at first chest CT and the age at initiation of dornase alfa (r = 0.658; p < 0.001). 

## DISCUSSION

In the present study we found a high frequency of early lung disease in young children with CF, with air trapping being the most prevalent finding, followed by mucoid impaction, bronchiectasis, and atelectasis. Only 4.4% of the chest CT scans were normal, corroborating data showing that structural lung disease is common in infants and preschool children with CF, even among those with mild clinical manifestations.^10^

Conventional chest X-ray has been traditionally used to monitor lung disease, although its sensitivity to detect early changes is limited.^2^ Although chest CT has been considered the gold standard for detecting and monitoring structural lung changes in patients with CF, its use in young children has been controversial because of exposure to ionizing radiation.^11^ In addition, previous studies of chest CT scans in young children with CF have shown inconsistent CT findings over time,^3^ ^,^ ^4^ and there are inherent difficulties in performing CT scans in young children, including the need for sedation. Despite these controversies, a recent Delphi consensus panel of CF specialists in Brazil reported that chest CT scans should be performed once every 2 years for monitoring stable patients.^12^ This approach would result in approximately nine chest CT scans from 1 to 17 years of age, an acceptable risk-benefit ratio regarding radiation exposure in the opinion of the panelists.^12^ Our findings of a high frequency of structural abnormalities at a median age of 3 years reinforce the panel recommendation that early, periodic imaging is an important tool for personalized treatment in Brazil. 

Regardless of timing, the decision to perform chest CT should consider the need for optimized protocols that provide maximum diagnostic sensitivity with the lowest possible dose. As highlighted in the Brazilian Delphi consensus panel, as well as in other studies, standardizing these protocols is critical to optimize the quality-to-dose ratio and allow image comparability among CF centers.^11^ ^-^ ^13^ Access to high-cost, radiation-free imaging modalities capable of detecting early lung disease in patients with CF-such as hyperpolarized Xenon magnetic resonance imaging-remains limited.^14^

Our data show that therapeutic decisions were strongly associated with radiological findings, with a clear association between the age at first chest CT scan and initiation of inhaled dornase alfa therapy. Although clear indications for initiation of dornase alfa in infants and preschool children with CF are almost nonexistent, data on the impact of inhaled 7% hypertonic saline in children in the 3- to 6-year age bracket have shown significant improvement in lung clearance index and structural lung disease after 48 weeks, attributed to decreased mucus viscosity, optimized mucociliary clearance, and potentially fewer exacerbations.^6^ ^,^ ^7^ ^,^ ^15^ Although inhaled hypertonic saline is used in our center, dornase alfa has been preferentially prescribed to CF patients in early life because of the difficulty in preparing hypertonic solutions at home and the lack of commercially available formulations in Brazil. 

The choice of mucolytic agent to be started early in life to prevent or reduce the extent of progressive CF lung damage could also be influenced by the results of small studies showing potential benefits of dornase alfa in other endpoints, such as pulmonary inflammation,^16^ lung function,^17^ and chest CT abnormalities.^18^ However, those data were obtained from studies of CF patients beyond early childhood, typically > 5-6 years of age. 

CFTR modulators constitute the most important advance in CF care.^19^ Although CFTR modulators are available for infants with CF in high-income countries, in Brazil they are approved for use in children ≥ 6 years of age only, underscoring the need for close monitoring and timely interventions in the younger population. 

The present study has several limitations that warrant consideration. The cross-sectional design precluded the evaluation of longitudinal lesion progression. The absence of a validated scoring system restricts comparability and limits the quantification of disease severity. A potential risk of bias related to the interpretation of chest CT images by many different professionals without formal standardization must be acknowledged. However, our data reflect real-world clinical practice, and the inclusion of interpretations from multiple professionals may increase external validity and reduce the influence of systematic bias from a single observer, although interobserver variability remains a limitation. Another limitation is the fact that we did not assess genotype, bacterial colonization patterns, or correlations with pulmonary function, all of which could provide further insight into the heterogeneity of early CF lung disease. 

Despite the aforementioned limitations, our results depict the care provided for infants and preschool children in a specialized CF center in Brazil, where the first chest CT scan was performed at a median age of approximately 3 years, revealing a high prevalence of early structural lung disease. A strong association was observed between the age at first CT and the initiation of dornase alfa therapy, indicating that imaging findings substantially influenced therapeutic decisions. By reflecting the subclinical, insidious, and progressive course of CF lung disease, these findings strongly reinforce the urgency of providing earlier access to CFTR modulators for Brazilian children with CF. 
